# Low immunogenic endothelial cells endothelialize the Left Ventricular Assist Device

**DOI:** 10.1038/s41598-019-47780-7

**Published:** 2019-08-05

**Authors:** Constanca Figueiredo, Dorothee Eicke, Yuliia Yuzefovych, Murat Avsar, Jasmin Sarah Hanke, Michael Pflaum, Jan-Dieter Schmitto, Rainer Blasczyk, Axel Haverich, Bettina Wiegmann

**Affiliations:** 1 Institute for Transfusion Medicine, Hannover Medical School, 30625 Hannover, Germany; 2 SPP2014 – Towards an implantable lung, Hannover, Germany; 3 Department of Cardiothoracic, Transplantation and Vascular Surgery, Hannover Medical School, 30625 Hannover, Germany; 4 Leibniz Research Laboratories for Biotechnology and Artificial Organs, Hannover Medical School, 30625 Hannover, Germany; 5 German Centre of Lung Research, 30625 Hannover, Germany; 6 REBIRTH Cluster of Excellence, Hannover Medical School, Hannover, Germany

**Keywords:** Biomaterials - cells, Molecular medicine

## Abstract

Low haemocompatibility of left ventricular assist devices (LVAD) surfaces necessitates anticoagulative therapy. Endothelial cell (EC) seeding can support haemocompatibility, however, the availability of autologous ECs is limited. In contrast, allogeneic ECs are readily available in sufficient quantity, but HLA disparities induce harmful immune responses causing EC loss. In this study, we investigated the feasibility of using allogeneic low immunogenic ECs to endothelialize LVAD sintered inflow cannulas (SIC). To reduce the immunogenicity of ECs, we applied an inducible lentiviral vector to deliver short-hairpins RNA to silence HLA class I expression. HLA class I expression on ECs was conditionally silenced by up to 70%. Sufficient and comparable endothelialization rates were achieved with HLA-expressing or HLA-silenced ECs. Cell proliferation was not impaired by cell-to-Sintered Inflow Cannulas (SIC) contact or by silencing HLA expression. The levels of endothelial phenotypic and thrombogenic markers or cytokine secretion profiles remained unaffected. HLA-silenced ECs-coated SIC exhibited reduced thrombogenicity. In contrast to native ECs, HLA-silenced ECs showed lower cell lysis rates when exposed to allogeneic T cells or specific anti-HLA antibodies. Allogeneic HLA-silenced ECs could potentially become a valuable source for LVAD endothelialization to reduce immunogenicity and correspondingly the need for anticoagulative therapy which can entail severe side effects.

## Introduction

According to the World Health Organization, cardiovascular diseases are the leading cause of death worldwide, with an increasing incidence and prevalence^1^. While death attributable to coronary artery disease has been halved since the 1980s, heart failure has almost tripled within the same time frame^2^. Treatment strategies for heart failure include optimized drug therapy, surgical resolution, and, ultimately, heart transplantation (HTx) as currently the only curative approach^3^. Due to an increasing disparity between the number of patients in need of a HTx and the number of potential organ donors, the implantation of left ventricular assist devices (LVAD) as a bridge to HTx has become the standard of care for patients with end-stage heart failure. Several studies have demonstrated that the outcome of patients who receive a LVAD is better the earlier it is implanted. Furthermore, LVAD implantation is increasingly being performed as a life-saving final destination therapy to improve the quality of life for recipients^4,5^.

Common to all LVADs, whether continuous or pulsatile flow LVADs, or first, second or third generation LVADs, are high rates of acute and chronic complications, caused by the inevitable contact between the artificial surfaces of the device, the circulating blood and the surrounding tissue. The surgical implantation of LVADs offers entry points for pathogens, which can result in local infection or severe sepsis. Anticoagulation therapy is similarly prone to complications – high doses can result in severe bleeding, and low doses in thrombosis, which, in turn, can lead to stroke or the need to replace the LVAD^4,6,7^. To address these complications and provide a secure and long-lasting destination therapy, ‘biologizing’ artificial materials with a suitable cell source offers a promising approach. As the native vascular endothelium presents a non-thrombogenic surface, the endothelialization of cardiovascular devices has been widely accepted as an efficient method to improve haemocompatibility of artificial surfaces^8^. Although, autologous endothelial cells (ECs) would be the best option, it is unlikely that autologous ECs harvested from critically ill patients could be expanded *ex vivo* to quantities sufficient for the endothelialization of a LVAD. Therefore, allogeneic ECs represent the only feasible alternative to produce the large quantities of cells required to cover the large device surfaces. However, ECs are highly immunogenic and disparities at the high variable human leukocyte antigen (HLA) loci and minor histocompatibility antigen (mHAg) may trigger potent immune responses that lead to EC rejection^9^. In previous work, we have developed a strategy to reduce the immunogenicity of cells using ribonucleic acid (RNA) interference and demonstrated that silencing HLA expression protects allogeneic cells against humoral and cellular responses *in vitro* and *in vivo*^10–13^.

Our aim in this study was to evaluate the general feasibility of endothelializing a LVAD. A biofunctionalized LVAD endothelialized with low immunogenic allogeneic ECs may improve haemocompatibility and therefore reduce or even eliminate the need for anticoagulative therapy. Successful proof of concept of this technology could pave the way for LVADs as a genuine alternative to heart transplantation and a final destination therapy.

## Results

### ECs seeded on sintered titanium oxide inflow conduits (SIC) were capable of supporting a viable monolayer with proper cell-to-cell contact

**Figure 1:**
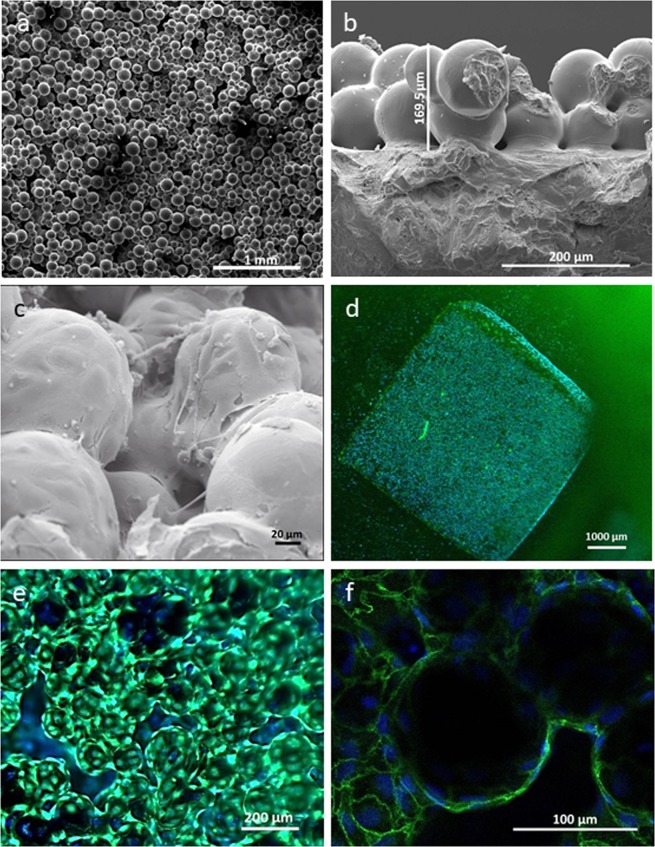
ECs form a sufficient and viable endothelial monolayer on SIC. (**a**,**b**) SEM indicated a variable spherical three-dimensional surface of the SIC. (**c**) The entire SIC surface is covered by ECs, which have modulated themselves on the spherical shapes of the sintered titanium oxide spheres. (**d**,**e**) Fluorescence staining using Calcein AM (green) and Hoechst 33342 (blue) revealed a viable and confluent endothelial monolayer on the SIC. (**f**) VE-cadherin staining of adherens junctions demonstrated the integrity of the endothelial monolayer (anti-VE-Cadherin = green, Hoechst 33342 = blue).

Scanning electron microscope (SEM) of native SIC revealed a three-dimensional surface with different distances between sintered titanium oxide spherules, and a variable height of 50 to 100 μm (Fig. 1a,b). The seeding protocol resulted in an endothelial monolayer on the SIC showing EC adherence to the spherules through the entire SIC surface (Fig. 1c,d). Calcein AM/Hoechst 33342 staining revealed a viable cell layer covering the seeded area (Fig. 1d to e). VE-cadherin staining confirmed the integrity of the EC monolayer on SIC (Fig. 1f). These data demonstrate the feasibility of endothelializing SIC with HLA class I-silenced ECs.

### ECs maintained their phenotype and functional reactivity to Tumor Necrosis Factor-α (TNFα) on SIC

**Figure 2:**
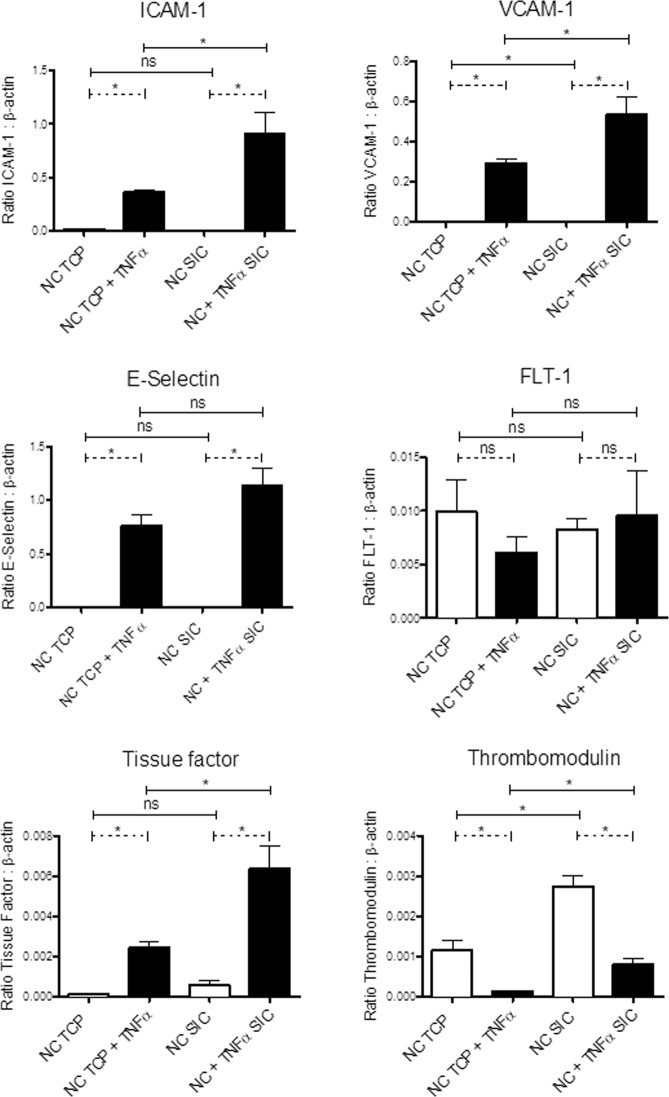
EC reactivity is comparable between TCP and SIC. Semi-quantitative real-time RT-PCR was used to determine mRNA levels, normalized to β-actin. Analysis of the endothelial phenotype (FLT-1), endothelial specific activation (ICAM-1, VCAM-1, E-Selectin) and thrombogenic state markers (tissue factor, thrombomodulin) of ECs on TCPs and SICs, with and without TNFα-stimulation indicated a comparable preservation of the biological reactivity of the ECs. The paired two-tailed t-test was used to determine statistically significant differences (p < 0.05) indicated by the asterisks (*). Statistically insignificant results are marked by “ns”. The results are depicted as group means ± standard deviation (n = 3).

Anti-inflammatory and anti-thrombogenic status are critical EC requirements for tissue engineering. Thus, expression levels of EC markers fms related tyrosine kinase-1 (FLT-1) as well as the endothelial specific activation intercellular adhesion molecule-1 (ICAM-1), vascular cell adhesion protein-1 (VCAM-1), E-Selectin and thrombogenic markers (thrombomodulin, tissue factor) were analyzed by semi-quantitative real-time reverse transcriptase polymerase chain reaction (RT-PCR) in ECs seeded on tissue culture plates (TCPs) or SIC in presence or absence of TNFα (Fig. 2). The expression levels of FLT-1 indicated a stable endothelial phenotype, which was not affected by TNFα-stimulation or by cell-to-SIC contact compared with TCPs. For all activation markers and tissue factors, TNFα-stimulation resulted in significantly higher expression levels in both TCPs and SICs compared with unstimulated ECs, and in a significant decrease in thrombomodulin. Comparing unstimulated ECs between both groups, significantly higher expression levels of VCAM-1 and thrombomodulin were detected for SICs. Similar observations were made using TNFα-stimulated ECs; significantly higher expression levels of ICAM-1, VCAM-1, tissue factor and thrombomodulin were identified for SIC in comparison to TCP (Fig. 2).

### Comparable seeding efficiency, cell proliferation and functional reactivity of non-silenced and HLA class I-silenced ECs on TCP and SIC

Fluorescence microscopy analysis also revealed a sufficient and viable short hairpin targeting β2-microglobulin (shβ2m)-expressing EC monolayer, covering the entire SIC surface, which was comparable to the levels of endothelialization achieved by non-silenced ECs (Fig. 1d,e).

**Figure 3:**
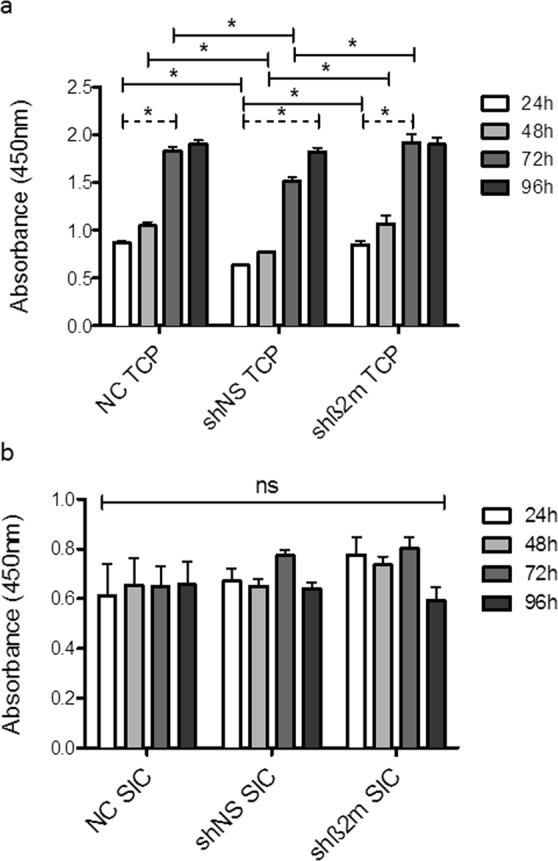
HLA class I silenced ECs are suitable for SIC endothelialization. (**a**) For each cell type on TCP, WST-8 assays results confirm a daily significant increase of the absorbance up to 72 h, until a steady state is reached on day four. (**b**) The seeding protocol of SICs resulted in a steady state of the absorbance from the outset without any significant differences between the cell types, indicating a stable and viable endothelial monolayer on SICs. The paired two-tailed t-test and the one-way ANOVA analysis were used to determine statistically significant differences (p < 0.05) as indicated by the asterisks (*).

The WST-8 assay was used to evaluate the proliferation and viability of non-silenced (NC), non-specific short hairpin (shNS)-expressing ECs and shβ2m-expressing ECs on TCP and SIC. The seeding protocol of the TCP resulted in a significant increase in the absorbance within each of the respective cell types up to 72 h; after this point, no significant increase was detected at 96 h, except for shNS. Furthermore, at each time point up to 72 h, a significant difference between shNS and NC, and between shNS and shβ2m was detected. At 96 h, no significant differences were identified between all three groups. In particular, we observed no significant differences between NC and shβ2m at any of the time points (Fig. 3a). For the SIC seeding protocol, no significant differences were detected between all three cell types at any of the time points. This is indicative of an efficient SIC seeding protocol, and confirms that a stable, viable endothelial monolayer was established at an early stage (Fig. 3b).

**Figure 4:**
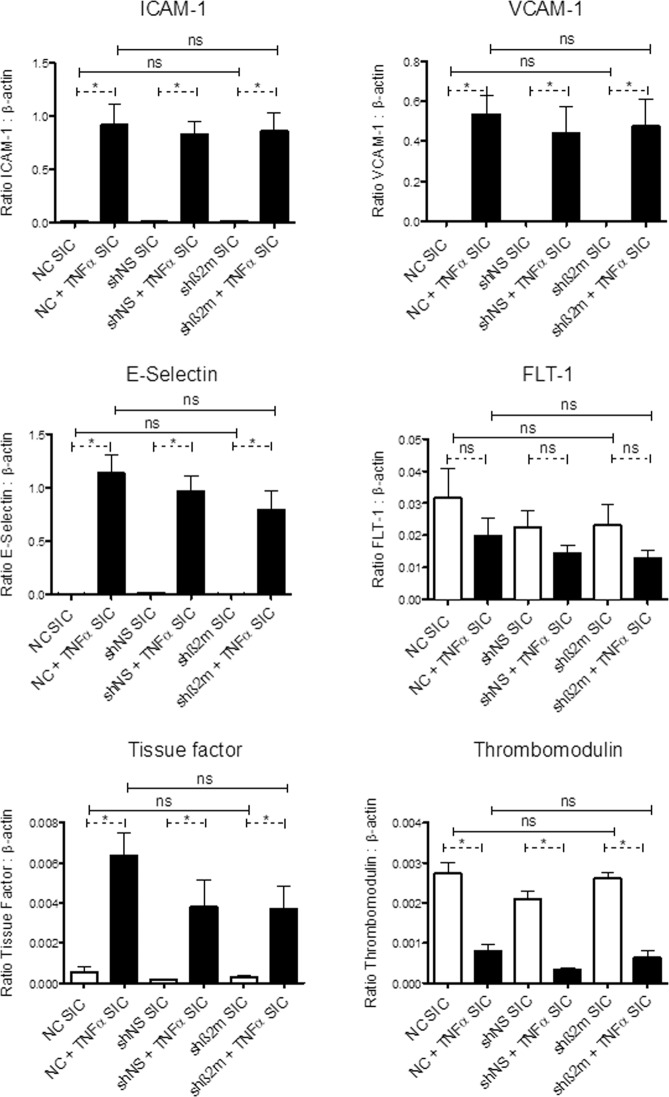
HLA class I-silenced ECs respond to external stimuli. RT-PCR analysis of the endothelial phenotype (FLT-1), endothelial specific activation (ICAM-1, VCAM-1, E-Selectin) and thrombogenic state markers (tissue factor, thrombomodulin) of NC, shNS and shβ2m on SICs, with and without TNFα-stimulation indicated a stable endothelial phenotype and a physiological response to TNFα-stimulation, which is not affected by the cell-to-SIC contact, or by silencing HLA-class I expression. The paired two-tailed t-test and the one-way ANOVA analysis were used to determine statistically significant differences (p < 0.05) indicated by the asterisks (*). Statistically insignificant results are marked as “ns”. The results are expressed as group means ± standard deviation (n = 3).

**Figure 5:**
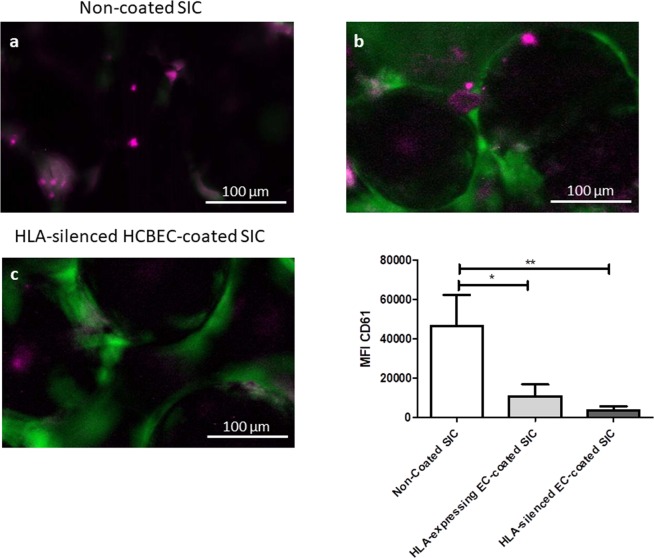
Lower thrombogenicity of endothelialized SIC compared to native SIC. Native or endothelialized SIC with HLA class I-expressing or silenced ECs were exposed to platelets, which were activated with ADP and thrombin for 1 h. Platelets were labeled with Allophycoyanin conjugated anti-CD61 antibody (magenta) and ECs expressed GFP as reporter gene (green). Thrombus formation was analyzed by fluorescence microscopy, indicating that (**a**) native SIC was more thrombogenic than (**b**) SIC endothelialized with HLA class I-expressing ECs or (**c**) SIC endothelialized with HLA class I-silenced ECs. (**d**) Mean fluorescence intensities are depicted in the bar graph. The paired two-tailed t-test was used to determine statistically significant differences (p < 0.05) indicated by the asterisks (*). The results are expressed as mean ± standard deviation (n = 3).

A comparative analysis of the transcript levels of adhesion molecules and thrombogenic markers revealed no significant differences between NC, shNS- and shβ2m-expressing ECs on SIC (Fig. 4) for these cell types. A steady endothelial phenotype, detected by stable transcript levels of FLT-1, was observed, which was not affected by HLA-class I silencing, or by TNFα stimulation. However, in each of the cell types, TNFα-stimulation led to a significant increase in the relative expression levels of the activation markers (ICAM-1, VCAM-1 and E-Selectin) and the pro-coagulative tissue factor, and a significant decrease in anti-coagulative thrombomodulin. As there were no significant differences between the three cell types with or without TNFα-stimulation, the data indicated that physiological reactivity towards the TNFα-stimulation of the shβ2m ECs remained unaffected (Fig. 4). Similarly, IL-1β and IL-8-secretion profiles in the supernatants of HLA-expressing or HLA-silenced ECs after SIC endothelialization were comparable (Table 1). Importantly, in contrast to non-endothelialized SIC, endothelialized SICs were demonstrated to be less thrombogenic than native SICs, as significantly less platelet adhesion (p < 0.05) was observed when platelets were exposed to HLA class I-silenced ECs-coated SIC in comparison with non-coated SIC. No significant differences in platelet adhesion were observed between SIC coated with HLA-expressing or HLA-silenced ECs (Fig. 5).

**Table 1:** Cytokine secretion profile.

pg/ml	NC	NC + IFN-γ	shNS	shNS + IFN-γ	shβ2m	shβ2m + IFN-γ
IL-1β	8.1 ± 6.3	831.6 ± 345.9	8.1 ± 6.3	735.8 ± 221.7	8.1 ± 6.3	924.5 ± 402.2
IL-8	21.1 ± 18.3	15,988.1 ± 1,637.3	19.0 ± 11.5	18,016.1 ± 2,449.1	23.7 ± 16.5	16,313.1 ± 1,650.6

### HLA class I silencing protects EC against immune responses

**Figure 6:**
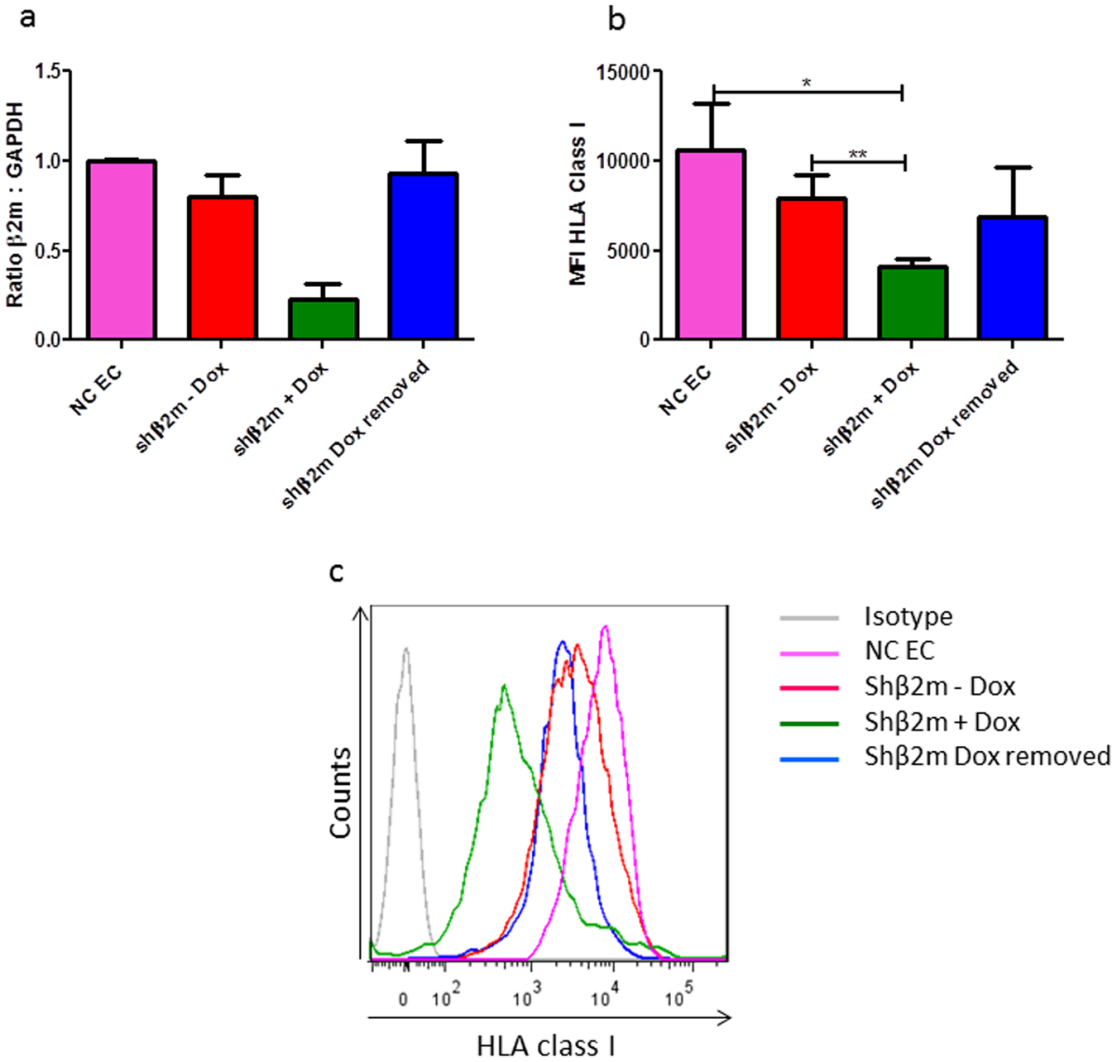
Regulation of endothelial HLA class I silencing by Doxycycline. ECs expressing shβ2m and the trans-activator tTR-KRAB were cultured in the presence or absence of 100 ng/ml doxycycline and after removal of doxycycline. Levels of β2-microglobulin transcripts (**a**) measured by real-time PCR were higher in non-modified ECs, transduced ECs in the absence of doxycycline (shβ2m – Dox) or after withdrawal of doxycycline (shβ2m Dox removed). (**b**) These results were confirmed at protein level as detected by flow cytometric analysis of HLA class I expression. The bar graphs represent mean values and standard deviations (n = 3). (**c**) Representative overlay of HLA class I expression in presence and absence of doxycycline.

ECs expressing shβ2m under the control of a Tet-ON promoter and a repressor protein showed a significant reduction of β2-microglobulin transcript levels and HLA class I protein levels upon addition of Doxycycline. Expression of HLA class I was restored after withdrawing Doxycycline. These data indicates the feasibility of conditionally silencing the expression of HLA class I on ECs (Fig. 6). Nevertheless, we have observed some leakiness, as the levels of HLA class I expression tend to decrease already in the absence of Doxycycline (Fig. 6a to c).

**Figure 7:**
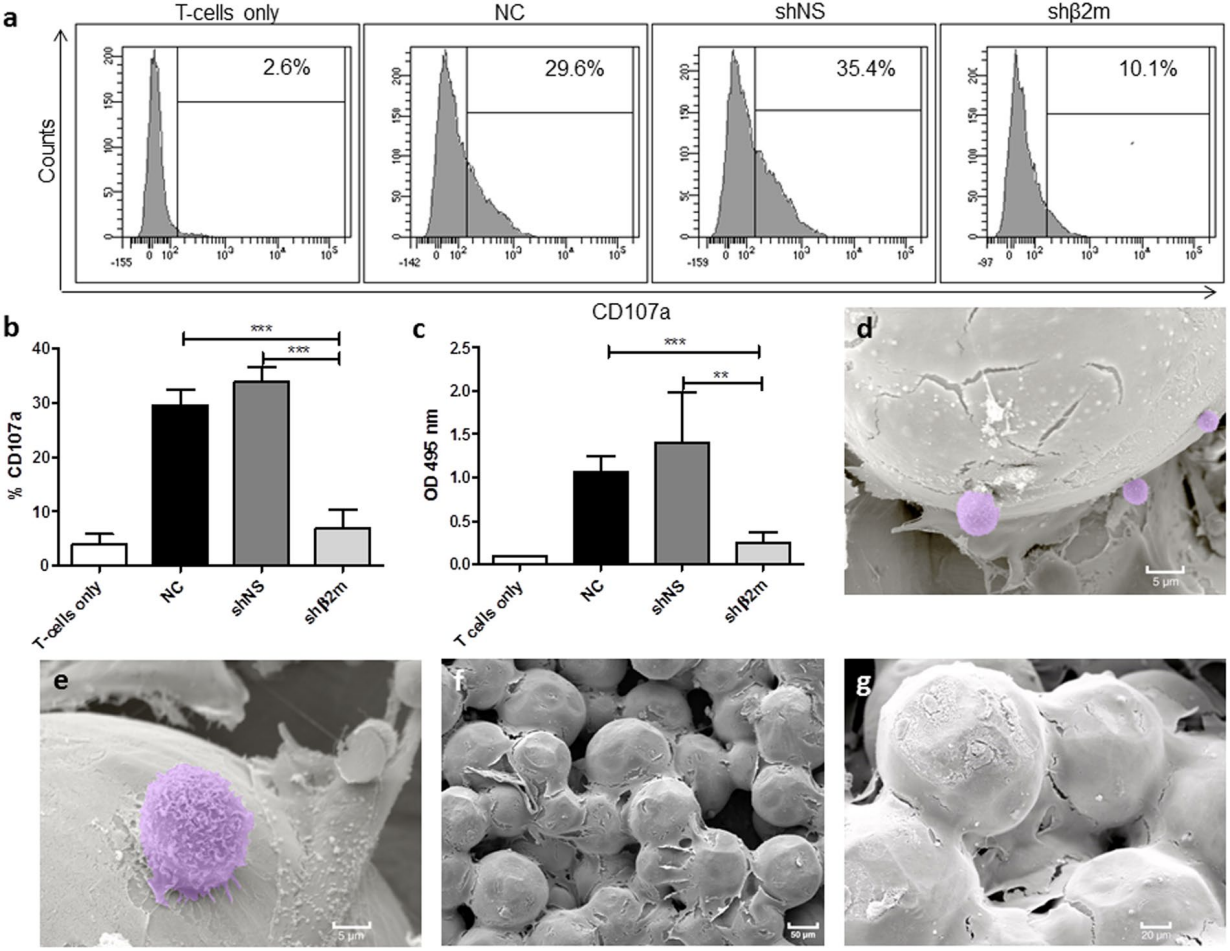
HLA class I silenced EC on SIC are protected against CD8+ T cell cytotoxicity. NC, shNS or shβ2m ECs were incubated with CD8+ T cells; *p < 0.05; **p < 0.01; ***p < 0.001. (**a**) Representative histograms of the frequency of CD107a expression show higher CD8+ T-cell degranulation after exposure to HLA expressing ECs in comparison to HLA class I-silenced ECs. (**b**) The graph displays mean values and standard deviations of CD107a expression frequencies (n = 5). (**c**) Similarly, in CD8+ T-cell cytotoxic assays, HLA class I- expressing EC cell lysis rates were significantly higher in comparison to HLA class I-silenced cells. The graph shows mean values and standard deviations of optical densities values indicating LDH activity released by ECs (n = 5). (**d**,**e**) SEM analysis of the T-cell cytotoxic assay on SIC indicated the adherence of primed CD8+ T-cells only to non-silenced endothelial cells. For better visualization, the CD8+ T-cells on the NC ECs were highlighted in purple, (**f** and **g**) SEM analysis of shβ2m ECs on SIC revealed the absence of CD8+ T-cells on the cell surfaces.

In addition, the survival capacity of HLA-silenced ECs mounted on SIC against allogeneic T cell responses was evaluated in degranulation and cytotoxic assays. In the presence of HLA class I-silenced ECs, T-cells showed significantly lower degranulation rates (p < 0.001) in comparison with HLA class I-expressing ECs. Accordingly, in contrast to non-modified ECs (p < 0.001) or ECs expressing shNS (p < 0.01), significantly decreased T-cell lysis rates were observed when T-cells were co-cultured with HLA class I-silenced ECs (Fig. 7a–c). These data were also confirmed by the scan electron microscopy (SEM) analysis of the respective T-cell cytotoxic assays (Fig. 7d–g).

**Figure 8:**
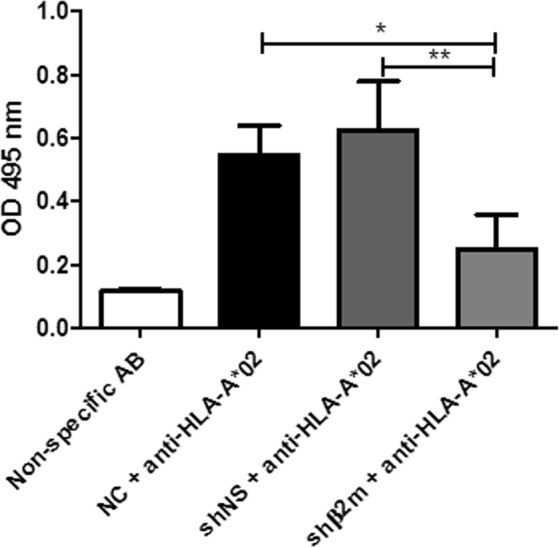
HLA class I-silenced EC on SIC are protected against antibody-mediated complement-dependent cytotoxicity. ECs typed for HLA-A*02 were used to coat SIC. NC, shNS or shβ2m ECs on SIC were exposed to specific anti-HLA-A*02 or non-specific anti-HLA-A*23 antibodies and complement. Levels of cell lysis, indicated by LDH activity, were measured by detecting optical densities (OD) at 450 nm. ECs expressing shβ2m showed significantly lower levels of LDH activity in comparison with NC and shNS, indicating that HLA class I-silenced EC on SIC are protected against antibody-mediated complement-dependent cytotoxicity. ANOVA was used to determine statistically significant differences indicated by the asterisks (*p < 0.05, **p < 0.01). The results are presented as mean ± standard deviation (n = 3).

In antibody-mediated complement-dependent assays, significantly lower lactate dehydrogenase (LDH) activity (OD 0.25 ± 0.11, p < 0.01) was detected in supernatants of HLA class I-silenced ECs in the presence of anti-HLA-A*02 antibodies and complement. In contrast, optical density (OD) values by up to 0.78 in the LDH assays were measured using supernatants of HLA-expressing ECs. No significant increase in LDH activity was detected when HLA-silenced ECs were incubated with the specific anti-HLA antibody in comparison with non-modified ECs incubated with a non-specific antibody (anti-HLA-A*23) used as control. These results indicate that HLA class I silencing also protects ECs against antibody-mediated complement-dependent cytotoxicity (Fig. 8).

## Discussion

Taking LVAD therapy towards a permanent, functionally safe and genuine alternative to heart transplantation means working to minimize the side effects of the therapy. Future developments must allow progress beyond technical down-sizing and flow-optimization^14^. Device optimization needs to focus on the ‘biologization’ of the LVAD system to create bio- and haemocompatible surfaces within a mechanically-enhanced biohybrid system. This may contribute to the minimization or even elimination of the use of anticoagulative therapy with its associated risks^14^.

As LVAD inflow cannulas are one of the areas most prone to the development of thrombus formation^15^, we began by endothelializing the SIC material (Supplementary Fig. 1) and were successful in achieving complete coverage with a viable EC monolayer. Importantly, VE-cadherin staining demonstrated sufficient cell-to-cell interactions of the ECs on the SIC, which are required for the maintenance of an intact monolayer, in particular during flow application^16^. For a long-term improvement with respect to the haemocompatibility of the endothelialized SIC, the seeded endothelial monolayer needs to display adequate physiological properties, presenting an anti-thrombogenic and anti-inflammatory surface^8^. Our transcript level analyses of ECs on SIC focused on endothelial-specific pro-inflammatory markers, such as ICAM-1, VCAM-1 and E-Selectin. In addition, we determined the levels of tissue factor, which enables the initiation of the coagulation cascade, and its counterpart thrombomodulin, involved in the anticoagulative pathway^17^. These studies confirmed the crucial anti-inflammatory and anti-thrombogenic status of ECs on SIC, which was not affected by silencing HLA expression or by cell-to-SIC contact, and was physiologically induced by TNFα-stimulation^18^.

Usually, ECs indicate a concurrent up-regulation of activation and thrombogenic state markers under shear-stress exposure^19,20^. This physiological feature was also observed for the ECs on the SIC, as they presented significantly higher expression levels of ICAM-1, VCAM-1, tissue factor and thrombomodulin compared with the ECs on TCP, which were caused by the applied shear stress within the rotational SIC seeding protocol. However, the up-regulation of the expression of those markers was significantly lower compared with their relative expression levels in TNFα-stimulated ECs on the TCPs and SIC. Nevertheless, they were responsible for the initially higher values of the ECs on the SIC, resulting conversely in higher expression levels after TNFα-stimulation^13,18–20^. Overall, the endothelial monolayer on the SIC generally met the requirements for the stepwise implementation of a sophisticated biohybrid LVAD system.

For further development, an appropriate cell source for the ‘biologization’ of the LVAD needs to be identified. Indeed, from an immunological perspective, autologous ECs would be the optimal cell source. However, several millions of ECs are required for complete LVAD endothelialization, which cannot be obtained from one single individual and, in particular, not from severely ill patients due to their impeded EC proliferation potential. Allogeneic ECs may be used as an alternative, but, without modification, they are highly immunogenic and may trigger potent immune rejection responses. Despite the possible HLA disparities between EC donor and patient, allogeneic ECs are able to express co-stimulatory molecules that are required for the activation of memory T-cells and induce Interleukin (IL) -2 production^21,22^. Thus, ECs have the potential to be the initiator of and to be targeted by an allogeneic immune response^23^. In this study, RNA interference was used to silence the HLA class I expression on allogeneic ECs, and thereby creating a condition of immunological invisibility^12,13^.

Similarly to HLA class I-expressing ECs, HLA class I-silenced ECs were capable of sufficient SIC seeding. In addition, HLA class I-silenced ECs maintained the typical endothelial phenotype and were capable of responding to extracellular stimuli such as TNF-α. Cytokine secretion profiles of ECs were analyzed as another relevant cellular parameter of functionality. In particular, we focused on the secretion of IL-1β and IL-8, which are known to play an important role within immune response^24,25^. The comparative analysis of non-silenced and HLA class I-silenced ECs indicated no significant differences between these two cell types on SICs. Overall, HLA class I-silenced ECs presented a resting but physiologically active endothelial monolayer on the SIC, which was not influenced either by silencing HLA expression or by the cell-to-SIC contact. Ultimately, HLA class I-silenced EC monolayer on the SIC contributed to reduced platelet adhesion and thrombus formation.

It is well known that cytotoxic CD8+ T-cell reactivity against allogeneic HLA class I molecules represent a major component of the cellular responses during EC rejection in organ and tissue transplantation. CD8+ T cells alone are also able to cause rejection of HLA class I-mismatched ECs^26–28^. In contrast to HLA class I-expressing ECs, HLA class I-silenced ECs on SIC were significantly less targeted by allogeneic T cells responses. NK cells are able to recognize and lyse cells with lower HLA class I expression on their surface, such as cancer cells or virus-infected cells^29^. In this study we demonstrated that the residual HLA-class I expression on the EC surface is sufficient to avoid natural killer (NK) cell activity against the HLA class I-silenced ECs (Supplementary Fig. 2). In addition, ECs used for the endothelialization of SIC are susceptible targets for pre- or de novo-formed antibodies. Antibodies specific for HLA may trigger complement-dependent cytotoxicity leading to EC lysis. Also, macrophages, neutrophils, and NK cells expressing receptors (FcγRs) for the Fc region of anti-HLA antibodies may trigger the recruitment of effector cells and cytotoxicity^30,31^. However, anti-HLA specific antibodies may also inhibit the expansion of regulatory T cells, which compromises graft tolerance^31,32^. The HLA class I-silenced ECs used to coat SIC were shown to be protected against antibody-mediated complement-dependent cytotoxicity. Thus, it can also be expected that HLA class I ECs on SIC will not be recognized by antibodies, leading to the recruitment of leukocytes and amplifying allogeneic immune responses.

A durable and sufficient HLA class I silencing effect was demonstrated in this study. However, HLA class I re-expression might be desirable in cases of viral infections or the development of cancer^33,34^. This could facilitate the recognition of affected allogeneic ECs and trigger an appropriate immune response leading to the elimination of the diseased ECs. Different strategies have been developed to generally regulate gene expression in a controllable manner, for example, by using Tetracycline-regulated systems^35^. A controllable silencing system of HLA expression on ECs could potentially provide a safety strategy in case of infection or cancer. By applying a doxycycline Tet-ON system, we demonstrated the feasibility of conditionally regulating the HLA class I expression on ECs. This approach may in the future have a wider application beyond ECs derived from human cord blood. The significance of a controllable HLA silencing system is even greater if allogeneic induced pluripotent stem cell (iPSC)-derived ECs are considered as a prospective cell source for endothelialization.

## Conclusion

In this study, we demonstrated the feasibility of endothelializing SIC with ECs. In particular, the use of low immunogenic ECs has the potential to simultaneously increase the haemocompatibility of SIC and decrease the strength of allogeneic immune responses. Interestingly, the genetic modification of ECs to downregulate HLA did not affect the capacity of ECs to respond physiologically to external stimuli. These results pave the way for a new era of biologized LVAD therapy, in which anti-coagulative therapy could be significantly minimized or omitted entirely. Working towards this aim, future experiments need to evaluate the flow resistance of the EC monolayer on SIC. Also, the development of ovine and porcine animal models to evaluate the efficiency of LVAD seeded with low immunogenic ECs will be an important milestone. Acute and chronic large animal models will be fundamental for the future evaluation of haematological, inflammatory and immunological responses targeting the EC monolayers on the SIC of a functional LVAD and can contribute towards optimizing LVAD function and therapeutic efficiency.

## Material and Methods

### Isolation and cell culture of human cord blood derived endothelial cells

ECs were isolated, initially cultured and characterized by fluorescence activated cell sorting (FACS) and RT-PCR as previously described^14,36^. Umbilical cords were obtained with informed consent. All experiments were performed in accordance with relevant guidelines, regulations and approved by the ethics committee of Hannover Medical School.

### Analysis of general possibility of endothelialization of sintered titanium oxide inflow conduits (SIC)

#### Endothelialization of sintered titanium oxide inflow conduit of HeartMate II

Sintered titanium oxide inflow conduits (SIC) taken from the HeartMate II (Thoratec, Pleasanton, California, USA) were cut in equally sized pieces (5 × 5 mm). SICs were placed in each 15 mL tube, separated by silicone plugs (Supplementary Fig. 1), filled with 15 mL endothelial growth medium (EGM-2, Lonza) and complemented with 5 × 10^6^ HLA class I–expressing ECs (NC). Two of these tubes were positioned in a circular glass container for four hours at rotational speed of 1 rpm on a Turning Device (Greiner, Frickenhausen, Germany) at 37 °C and 5% CO_2_. Endothelialized SICs were then transferred to 12-well tissue culture plates (TCP).

#### Qualitative analysis of SIC endothelialization

Visualizations of native and endothelialized SIC were conducted using scanning electron microscopy (SEM) (Philips SEM 505) as previously described^13^. Cell viability and seeding efficiency was evaluated by fluorescence microscopy (Axio observer A1 microscope, Zeiss, Germany) after EC staining with calcein acetomethylester (1 µg/mL, Calcein AM,Thermo Fisher) and trihydrochloride trihydrate (0.5 µg/mL, Hoechst 33342, Thermo Fisher) was performed. Immunofluorescence staining was used to illustrate the integrity of the cell-to-cell contacts within the EC monolayer on the SIC.

#### Gene expression analysis by real-time RT-PCR of ECs on TCP vs. SIC

To clarify whether endothelialization on SIC has a general impact on the gene expression profiles of ECs, NC were cultivated on TCP to form a confluent endothelial monolayer and cultivated on SICs for comparison. Half of the ECs were stimulated with tumor necrosis factor alpha (TNF-α) for 6 h (10 ng/mL, Bachem) and half were untreated. RNA isolation was performed using RNeasy mini Kit (Qiagen). Reverse transcription was done using the RevertAid^TM^ H Minus First Strand cDNA Synthesis Kit (Fermentas, Germany) using Random Hexamer Primers. Real-time RT-PCR was performed using Absolute SYBR Green Mix (ABgene). The applied primer pairs are described in the supplementary section (Supplementary Table 1). The data of the RT-PCR were analyzed using the 2^−ΔΔCT^-method using β-Actin as the housekeeping gene.

### Silencing HLA class I expression

#### Lentiviral constructs, vector production and lentiviral vector transduction

A conditional lentiviral vector system was used to deliver short-hairpin RNA (shRNA) to silence β2-Microglobulin expression. The vector pLVTHm, encoding for the shRNA, the green fluorescence protein (GFP) as reporter gene, and a vector encoding for tTR-KRAB trans-activator were used. The vectors were produced as previously described^13^. Lentiviral vector transduction was performed in six well TCPs. For this, 3 × 10^5^ ECs were seeded per well and infected with both vectors in presence of 8 µg/mL protamine sulfate (Sigma-Aldrich, Steinheim, Germany). After 16 h, ECs were washed with fresh culture medium. Transduction efficiency was calculated by the percentage of GFP-expressing cells assessed by FACS.

### Validation of application of silenced ECs for SIC endothelialization

#### Qualitative analysis of SIC endothelialization using silenced ECs

In accordance with the seeding protocol, β2-microglobulin silenced ECs (shβ2m) were used for the endothelialization of the SICs. Viability and qualitative seeding efficiency were analyzed by fluorescence microscopy.

#### Comparative quantitative analysis of EC growth on SIC

The proliferation potential between all three EC types, HLA class I-expressing (NC), non-specific shRNA (shNS) and shβ2m, was compared. This was analyzed under standard culture conditions in six well TCPs, for which 8 × 10^4^ ECs were applied initially, and, in a second step, analyzed on SICs, after finalizing the seeding protocol. The respective proliferation and viability of the ECs were measured every 24 h up to 96 h using the WST-8 Quick Cell Proliferation Assay Kit II (BioVision) as previously described^13^.

#### Comparative gene expression analysis by semi-quantitative real-time RT-PCR of the NC, shNS- and shβ2m-expressing ECs on SICs

To analyze whether the silencing procedure had an impact on the gene expression profile, SICs were seeded with NC, shNS and shβ2m ECs. Again, half of the ECs were stimulated with TNF-α for 6 h, while the other half remained unstimulated.

#### Assessment of cytokine secretion

The cytokine secretion profiles of all three cell types, NC, shNS and shβ2m ECs, after SIC endothelialization, were characterized using Luminex technology. For this, the ECs were cultured in presence or absence of IFN-γ (50 ng/ml) (Peprotech) for 48 h, following which supernatants were collected and analyzed for the secretion of IL-1β and IL-8.

#### Analysis of thrombogenicity potential of SIC

The thrombogenicity of SIC was analyzed by incubating activated platelets with uncoated SIC or coated with activated HLA-expressing or HLA class I-silenced ECs. Platelets (PLT) isolated by aphaeresis were stained with an allophycocyanin-conjugated anti-CD61 antibody. After 15 min, platelets were washed and resuspended in PBS. Furthermore, ECs were stimulated using TNF-α and platelets were stimulated using 2 U/ml Thrombin (T8885-10VL, SIgma-Aldrich) and 1 mM adenosine 5′-diphosphate sodium salt (ADP) (A2754, Sigma-Aldrich). 25 µl of stimulated PLTs were added to uncoated or endothelialized SIC after exposure to TNF-α for 1 h. Afterwards, SICs were washed with PBS to remove non-bound PLTs, following which the adhered PLTs and thrombus formation were analyzed by fluorescence microscopy.

### Validation of silenced ECs on SICs escaping immune response

#### Conditional HLA-class I silencing of ECs on SIC

ECs transduced for the expression of shRNA and the tTR-KRAB trans-activator were cultured in the presence and absence of 100 ng/ml Doxycycline (Sigma-Aldrich), respectively. β2-microglobulin transcript levels were evaluated by real-time RT-PCR. The native or genetically modified ECs were harvested and total RNA was isolated using the RNeasy Mini Kit (Qiagen, Hilden, Germany) according to the manufacturer’s instructions. Reverse transcription of RNA into cDNA was performed using the high-capacity cDNA reverse transcription kit (Applied Biosystems, Darmstadt, Germany). β2m transcript levels were analyzed by real-time PCR using specific predesigned TaqMan Gene Expression Assays. Expression values were calculated as ratio to β-actin, which was used as an internal control. All measurements were performed in triplicate. Relative quantification of β2m levels were performed using the 2^−ΔΔCT^ method. HLA class I expression levels were detected by FACS analysis upon staining the cells with an APC-conjugated anti-HLA class I antibody (w6/32, Serotec), respectively.

#### T- cell cytotoxic assays

CD8+ T-cells were isolated from peripheral blood collected from healthy individuals using the pan T-cell isolation kit (Miltenyi Biotec). Priming was performed by incubating CD8+ T-cells with donated, non-manipulated ECs for eight days in presence of 100 U/ml IL-2, 100 ng/ml IL-7 and 50 ng/mL IL-12 in (all Peprotech) culture medium supplemented with 5% AB serum (C.C. Pro). T-cell cytotoxic assays were then performed by the incubation of primed T-cells with SICs endothelialized either with NC, shNS or shβ2m ECs for 5 h in presence of 100U IL-2. The expression of CD107a on CD8+ T-cells was determined by FACS analysis upon staining with a PE-conjugated anti-CD107a antibody (BioLegend, London, UK). Cell lysis rates were evaluated by quantification of the activity of lactate dehydrogenase (LDH) in the supernatant released from damaged cells. LDH activity was determined using the Pierce LDH Cytotoxicity Assay Kit (Thermo Fisher) according to the manufacturer’s instructions. Additionally, SEM was performed to visualize CD8+ T-cells on endothelialized SIC.

#### Antibody-mediated complement-dependent cytotoxic assays (CDC)

The levels of antibody-mediated complement-dependent cytotoxicity (CDC) against ECs-coated SICs were determined. Only ECs typed for HLA-A*02 were used. SICs endothelialized with NC, shNS or shβ2m ECs were incubated with 1 µl of complement-binding specific and non-specific anti-HLA antibodies (OneLambda, Canoga Parc, CA) diluted 1:10 with PBS. 1 µl of PBS or anti-HLA-A*23 antibody (OneLambda) were used as negative controls and 1 µl of control-HLA positive reagent (Bio-Rad, Dreieich, Germany) was used as a positive control. Trays were incubated for 30 min at RT, followed by the addition of 5 µl of rabbit complement (Bio-Rad). After 1 h, cell lysis rates were determined by measuring LDH activity as described above.

### Statistical analysis

All tests were run in triplicate. The results were expressed as mean values with standard deviation (SD). Statistical analysis between two groups were performed using the unpaired two-tailed T-test and between multiple groups using the one-way ANOVA followed by Tukey’s Multiple Comparison Test (Prism 5.0 for Mac OS X, Version 5.0a, GraphPad Software, San Diego, CA, USA). A p-value of <0.05 was considered as a statistically significant difference.

### Ethics approval and consent to participate

This study was approved by the ethics committee of Hannover Medical School.

## Supplementary information

Supplementary Information

## Data Availability

All data is included in the manuscript.
