# Supplementary material for: Low immunogenic endothelial cells endothelialize the Left Ventricular Assist Device
Source: Sci Rep. 2019 Aug 5;9:11318. doi: 10.1038/s41598-019-47780-7 (PMC6683293; doi:10.1038/s41598-019-47780-7)
Supplement: Supplementary file 1 — Supplementary Information [file 41598_2019_47780_MOESM1_ESM.docx]

**Low immunogenic endothelial cells endothelialize the Left Ventricular Assisted Device**

Constanca Figueiredo^1,2,6^, Dorothee Eicke^1, 5^, Yuliia Yuzefovych^1,2,6^,Murat Avsar^3^, Jasmin Sarah Hanke^3^, Michael Pflaum^4^, Jan-Dieter Schmitto^3^, Rainer Blasczyk^1, 6^, Axel Haverich^2-6^, Bettina Wiegmann^2-6^

^1^ Institute for Transfusion Medicine, Hannover Medical School, 30625 Hannover

^2^ SPP2014 – Towards an implantable lung

^3^ Department of Cardiothoracic, Transplantation and Vascular Surgery, Hannover Medical School, 30625 Hannover, Germany

^4^ Leibniz Research Laboratories for Biotechnology and Artificial Organs, Hannover Medical School, 30625 Hannover, Germany

^5^ German Centre of Lung Research, 30625 Hannover, Germany

^6^ REBIRTH Cluster of Excellence, Hannover Medical School, Hannover, Germany

Emails:

[Figueiredo.Constanca@mh-hannover.de](mailto:Figueiredo.Constanca@mh-hannover.de) – corresponding author

[Eicke.Dorothee@mh-hannover.de](mailto:Eicke.Dorothee@mh-hannover.de)

Yuzefovych.Yuliia@mh-hannover.de

[Avsar.Murat@mh-hannover.de](mailto:Avsar.Murat@mh-hannover.de)

[Pflaum.Michael@mh-hannover.de](mailto:Pflaum.Michael@mh-hannover.de)

[Schmitto.Jan@mh-hannover.de](mailto:Schmitto.Jan@mh-hannover.de)

[Blasczyk.Rainer@mh-hannover.de](mailto:Blasczyk.Rainer@mh-hannover.de)

[Haverich.Axel@mh-hannover.de](mailto:Haverich.Axel@mh-hannover.de)

[Wiegmann.Bettina@mh-hannover.de](mailto:Wiegmann.Bettina@mh-hannover.de) – corresponding author

Running title: Biofunctionalized LVAD with low immunogenic endothelial cells

**Keywords:** Endothelium, Cell Therapy, Gene Therapy, Gene Expression and Regulation, Heart Failure**Supplementary Information**

**Methods**

**Immunofluorescence Staining**

Endothelialized SIC were fixed in 4% paraformaldehyde, followed by another washing step with phosphate-buffered saline (PBS). Then, the samples were incubated with 0.25% Triton-X100 diluted in Tris-buffered saline supplemented with 5% serum of the respective host of the secondary antibody to permeabilize and block the cells for unspecific antibody binding. After PBS washes, samples were incubated with the primary antibody anti-hVE-Cadherin (AbD Serotec, Germany). Repeated washing steps were performed, before dark incubation using fluorescence-labeled secondary antibodies goat anti-mouse Cy2 (Jackson ImmunoResearch, USA) was performed. After PBS washes, cell nuclei were stained using trihydrochloride trihydrate, followed by repeated washes. Finally, the samples were embedded in mounting medium (Immumount, DAKO, Germany) before fluorescence microscopic analysis was performed. As negative controls, samples were incubated with isotype-matching antibodies. For better visualization of the SIC, 3D-surface image series were taken and collated in a short video clip using the confocal microscopy (Leica TCS SP8, Leica microsystems, Germany).

**Natural Killer (NK) cell cytotoxic assays**

Natural killer (NK) cells were isolated using the NK cell isolation kit (Miltenyi Biotec). NK cell cytotoxic assays were performed using SICs endothelialized either with NC, shNS or shß2m ECs. NK cytotoxic assays were performed by exposing NK cells to the different ECs-coated SICs for 5h. Levels of cell lysis were detected by measuring LDH activity.


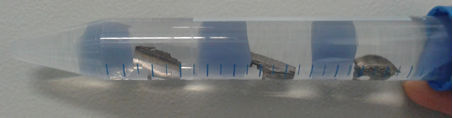


**Supplementary figure 1:** Three SIC (★) were placed in a 15mL tube, separated by silicone plugs and filled with ECs in EGM-2. Following, two of these tubes were positioned in a circular glass container for the 4h of application of the rotational speed.

**Supplementary Figure 2. HLA class I silenced HCBECs mounted on SIC are protected from NK cell cytotoxicity.**

Non-manipulated ECs or expressing shNS or shβ2m were incubated with NK cells isolated from peripheral blood. The bar graph represents means and standars deviations optical densities (OD) values indicating activity of LDH released by damage ECs in NK cell cytotoxic assays. No significant differences in NK cell cytotoxic activity were observed by using HLA class I –expressing or silenced ECs

**Supplementary table 1:** Primer pairs used for quantitative real-time RT-PCR.

| **Molecule** | **5´-Primer** | **3´-Primer** | **Amplicon [bp]** | **Annealing temperature [°C]** |
| --- | --- | --- | --- | --- |
| ß-Actin | ATT GCC GAC AGG ATG CAG AA | GGG CCG GAC TCG TCA TAC TC | 176 | 61 |
| ICAM-1 | CTA CCT CTG TCG GGC CAG GA | AGG CCT GCA GTG CCC ATT AT | 132 | 61 |
| VCAM-1 | GGC GCC TAT ACC ATC CGA AA | GAG CAC GAG AAG CTC AGG AGA A | 156 | 61 |
| E-Selectin | ATC CAG CCA ATG GGT TCG TG | GAA GGC TCT GGG CTC CCA TT | 114 | 61 |
| FLT-1 | ATC ATT CCG AAG CAA GGT GTG AC | TCC TTC TAT TAT TGC CAT GCG CT | 122 | 51 |
| Tissue Factor | CCC GAA CAG TTA ACC GGA AGA | GGA GTT CTC CTT CCA GCT CTG C | 191 | 61 |
| Thrombomodulin | GCC CAT GGG AGC TGG TTA GA | GGC CTG ACT TGG CCT GCT AC | 190 | 61 |
